# Precision Non-Alcoholic Fatty Liver Disease (NAFLD) Diagnosis: Leveraging Ensemble Machine Learning and Gender Insights for Cost-Effective Detection

**DOI:** 10.3390/bioengineering11060600

**Published:** 2024-06-12

**Authors:** Azadeh Alizargar, Yang-Lang Chang, Mohammad Alkhaleefah, Tan-Hsu Tan

**Affiliations:** 1 Department of Electrical Engineering, College of Electrical Engineering and Computer Science, National Taipei University of Technology, Taipei 10608, Taiwan; azadeh.tw@gmail.com (A.A.); ylchang@mail.ntut.edu.tw (Y.-L.C.); muhai@ntut.edu.tw (M.A.); 2 Innovation Frontier Institute of Research for Science and Technology, National Taipei University of Technology, Taipei 10608, Taiwan

**Keywords:** machine learning techniques, NAFLD, ensemble model, HSI, AUC, SMOTEENN

## Abstract

Non-Alcoholic Fatty Liver Disease (NAFLD) is characterized by the accumulation of excess fat in the liver. If left undiagnosed and untreated during the early stages, NAFLD can progress to more severe conditions such as inflammation, liver fibrosis, cirrhosis, and even liver failure. In this study, machine learning techniques were employed to predict NAFLD using affordable and accessible laboratory test data, while the conventional technique hepatic steatosis index (HSI)was calculated for comparison. Six algorithms (random forest, K-nearest Neighbors, Logistic Regression, Support Vector Machine, extreme gradient boosting, decision tree), along with an ensemble model, were utilized for dataset analysis. The objective was to develop a cost-effective tool for enabling early diagnosis, leading to better management of the condition. The issue of imbalanced data was addressed using the Synthetic Minority Oversampling Technique Edited Nearest Neighbors (SMOTEENN). Various evaluation metrics including the F1 score, precision, accuracy, recall, confusion matrix, the mean absolute error (MAE), receiver operating characteristics (ROC), and area under the curve (AUC) were employed to assess the suitability of each technique for disease prediction. Experimental results using the National Health and Nutrition Examination Survey (NHANES) dataset demonstrated that the ensemble model achieved the highest accuracy (0.99) and AUC (1.00) compared to the machine learning techniques that we used and HSI. These findings indicate that the ensemble model holds potential as a beneficial tool for healthcare professionals to predict NAFLD, leveraging accessible and cost-effective laboratory test data.

## 1. Introduction

Non-Alcoholic Fatty Liver Disease (NAFLD) is a prevalent liver condition with a significant worldwide impact, posing a substantial risk for the development of cirrhosis and hepatocellular carcinoma (HCC) [1]. Its prevalence affects around one-fourth of the global population [2], leading to considerable morbidity, increased mortality rates, and imposing a significant burden on affected individuals, their families, and healthcare systems [3]. Remarkably, the prevalence of NAFLD in Asian countries is comparable to that in Western countries [2,4]. NAFLD is characterized by the abnormal accumulation of fat in liver cells (hepatocytes) and is increasingly recognized as a major public health concern due to its high prevalence worldwide. The rise in obesity and metabolic syndrome contributes to NAFLD becoming the leading cause of chronic liver disease and abnormal liver function tests in China [2,5,6]. Recently, NAFLD has emerged as the primary cause of chronic liver disease and the fastest-growing indication for liver transplantation [2,3,7].

Liver biopsy has long been regarded as the gold standard for diagnosing Non-Alcoholic Fatty Liver Disease (NAFLD). However, this diagnostic method has several drawbacks that limit its routine use in public health check-up studies [8,9]. The procedure is invasive, posing a risk of bleeding, and may not be suitable for widespread application. To address these concerns, alternative diagnostic techniques have been explored, such as ultrasonography, magnetic resonance imaging, and computed tomography, which can also detect NAFLD. However, these imaging methods come with their own limitations, including being time-consuming, expensive, and often not easily accessible, particularly in remote regions. Although abdominal ultrasound has been widely used for the assessment of NAFLD, while ultrasound is a non-invasive and relatively affordable imaging technique, it does have some limitations when applied to NAFLD diagnosis and characterization. One of the primary challenges lies in its sensitivity to detecting mild levels of fat infiltration, often resulting in false negatives in cases of early stage NAFLD. The presence of obesity can also hinder the clarity of ultrasound images, making it challenging to accurately assess liver fat content and inflammation. Abdominal ultrasound for NAFLD presents additional complexities that demand specialized expertise. Interpreting ultrasound images for NAFLD requires skilled radiologists or sonographers familiar with the specific nuances of liver appearance associated with fatty infiltration. However, not all regions have access to such specialized medical professionals, exacerbating the diagnostic challenge [10,11,12]. Due to the invasiveness and cost associated with a liver biopsy, it is not routinely performed, prompting the need for non-invasive and more practical diagnostic approaches to effectively identify and manage NAFLD [13].

Recent advancements in the field of Non-Alcoholic Fatty Liver Disease (NAFLD) have led to the development of several new indices for its diagnosis and assessment, including the hepatic steatosis index (HSI) and fatty liver index (FLI). These novel indices integrate various non-invasive parameters, such as clinical, biochemical, and imaging data, to offer a more comprehensive and accurate evaluation of NAFLD. Unlike traditional methods that require invasive liver biopsies, these new indices provide a safer and less burdensome approach to diagnosing and monitoring NAFLD. These innovative indices can be easily implemented in routine clinical practice, enabling earlier detection, intervention, and improved management of NAFLD to mitigate its potential complications. As research continues in this area, FLI and HSI, and other emerging indices hold great potential to revolutionize the diagnosis and management of NAFLD, ultimately contributing to better patient outcomes and reducing the disease’s global burden [5,14,15].

HSI is a non-invasive index used to assess the presence of hepatic steatosis, also known as fatty liver disease. It is based on easily obtainable clinical and biochemical parameters, making it a practical tool for diagnosing and monitoring NAFLD. HSI incorporates factors such as body mass index (BMI), and the levels of certain liver enzymes, including aspartate aminotransferase (AST) and alanine aminotransferase (ALT). The index is calculated using a specific formula. The simplicity and effectiveness of HSI make it a valuable screening tool, particularly in settings where more sophisticated imaging or liver biopsy options might be limited. However, like other non-invasive indices, HSI may not provide a definitive diagnosis and is often used in conjunction with other diagnostic tools to assess the severity and progression of hepatic steatosis. Ongoing research and validation studies are further refining the utility of HSI in clinical practice, offering a valuable contribution to the management of NAFLD.

If indices utilized for NAFLD diagnosis, including HSI, showcase varying degrees of accuracy and area under the curve (AUC), it underscores an ongoing requirement to explore innovative avenues for enhancing early NAFLD detection with heightened precision. A notable drawback of these indices lies in their limited potential to consistently achieve this goal. This drawback accentuates the urgency for more sophisticated and precise methodologies, such as employing machine learning techniques, to refine NAFLD prediction by encompassing a wider array of clinical and biochemical variables. Machine learning harnesses advanced algorithms to scrutinize intricate patterns and relationships within extensive datasets, thereby fostering the development of more refined and personalized diagnostic models. By integrating diverse clinical, imaging, and biochemical data points, machine learning algorithms can heighten the precision of identifying hepatic steatosis, thereby contributing to the early recognition of individuals at risk. This proactive strategy holds significant promise in enhancing patient outcomes by enabling timely interventions and tailored management strategies.

The field of medicine is abundant with data, yet physicians may inadvertently overlook crucial information necessary for accurate disease diagnosis and treatment. Machine learning techniques, widely employed across diverse areas of health sciences, offer a potential solution to this problem. Leveraging large datasets, these techniques can effectively address the challenges of information extraction and analysis. In fact, machine learning has already been successfully utilized in numerous medical disciplines for disease prediction. Predicting NAFLD plays a crucial role in early detection, resource allocation, and public health planning, ultimately leading to improved management. However, the available data on the use of machine learning models for NAFLD prediction worldwide have been limited.

Machine learning models have been employed for several years to predict NAFLD [16,17,18]. Weidong Ji et al. [16] employed four machine learning algorithms to predict NAFLD in 304,145 adults, with XGBoost showing the best accuracy (0.880) and AUC (0.951). Xu et al. [17] used 11 techniques on a dataset of 2,522 individuals to achieve an 83% accuracy for NAFLD prediction. Liu et al. [18] found XGBoost to have the highest accuracy (0.795) among seven models for diagnosing NAFLD in 15,315 Chinese subjects.

The aim of our study is to develop an ensemble machine learning model for the prediction of NAFLD and compare its performance with HSI, using laboratory tests that are easily obtainable and cost-effective. By utilizing such data, we seek to enhance the accuracy and performance of NAFLD prediction, which could have important implications for both diagnosing and treating NAFLD.

## 2. Materials and Methods

This research adopts a comprehensive approach to develop an ensemble machine learning model to predict NAFLD, encompassing several key steps. Firstly, the datasets underwent thorough review and preprocessing. Next, feature ranking was performed. Subsequently, multiple machine learning models were developed and applied. The performance of each model was evaluated utilizing various metrics such as F1 score, precision, accuracy, recall, confusion matrix, receiver operating characteristics (ROC), AUC, and the mean absolute error (MAE). Based on the results, the most effective models for predicting NAFLD were determined. Subsequently, a novel ensemble machine learning model was introduced. Additionally, HSI was calculated and compared, and its performance was evaluated using the same set of metrics. Lastly, the two evaluated results were compared to determine their relative performance. These models were developed using the Python programming language on Google Colab (Colaboratory).

### 2.1. Dataset Description

This study utilized the National Health and Nutrition Examination Survey (NHANES) dataset, administered by the Centers for Disease Control (CDC) of the United States [19]. The dataset comprises 2505 subjects. Our focus was on 23 specific relevant features that are crucial for NAFLD investigation. These features were meticulously selected based on their potential significance in improving our understanding of NAFLD. Notably, the selected features include laboratory tests levels of Total Cholesterol, Weight, Height, BMI, Waist Circumference, white blood cell count (WBC), red blood cell count (RBC), systolic blood pressure (SBP), diastolic blood pressure (DBP), Hemoglobin, albumin, alkaline phosphatase (ALP), aspartate aminotransferase (AST), alanine aminotransferase (ALT), *γ*-glutamyl transferase (GGT), Creatinine, Triglycerides, LDL, HDL, and Fasting Glucose. Furthermore, demographic variables comprising age and gender, alongside the presence of NAFLD were included.

### 2.2. Preprocessing Data and Feature Ranking

To ensure the accuracy of our diagnosis models and disease predictions, it was essential to use procedure of data cleaning. Before analysis, the dataset used in this study contained instances of duplicate and missing data. A median imputation approach was employed for handling missing data. This method is widely accepted and effective in addressing missing data with random missing data. Using the median has advantages like being less affected by outliers and being a reliable estimate. Additionally, median imputation is straightforward, robust, and commonly utilized in various medical research. The median imputation method holds the original distribution of the data while effectively mitigating the influence of missing values on the analysis outcomes. Earlier studies have demonstrated that it surpasses other methods in enhancing analysis accuracy and reducing bias. So, median imputation was deemed suitable for this study because it is effective in handling missing data and can improve the quality of our findings.

After handling missing data, removing duplicates, and converting certain string features to numeric, the datasets underwent data standardization. Following that, the Synthetic Minority Oversampling Technique Edited Nearest Neighbors (SMOTEENN) was applied to tackle the issue of imbalanced data. Given the imbalance between the majority and minority classes in classification problems, the majority class often has a significantly larger number of examples compared to the minority class, leading to biased models. SMOTEENN combats this by generating synthetic samples of the minority class using SMOTE and subsequently cleaning the samples using ENN. This approach creates a more balanced dataset and reduces bias in machine learning models, enhancing the representation of the minority class.

In the next step, feature importance analysis was performed to assess the predictive efficacy of input attributes and to prioritize them accordingly. This study utilized embedded methods, a category of feature importance techniques. These methods integrate feature selection into the model training process, assigning weights or coefficients to individual features based on their importance. Features with greater coefficients or weights are considered more significant. By incorporating feature selection during model training, embedded methods select the most relevant features, contributing to improved model accuracy.

To address leakage, which is a problem in machine learning studies [20], SMOTEENN was exclusively applied to the training set after the dataset was split. This adjustment was made to ensure the absence of potential data leakage and to ensure the robustness of our model evaluation.

### 2.3. HSI Calculations

HSI is a validated predictive index developed by Lee et al. in 2009 for determining the presence of NAFLD. This index employs the parameters of body mass index (BMI), ALT/AST ratio, and the presence of diabetes or female gender, to calculate the likelihood of NAFLD, as indicated by the following formula [21,22,23,24]:

HSI = 8 alanine aminotransferase/aspartate aminotransferase ratio + body mass index (BMI) + 2 if diabetes + 2 if female

### 2.4. Model Selection

To predict NAFLD and facilitate early diagnosis and treatment, this study utilized affordable and easily accessible laboratory test data. An approach in machine learning for medical diagnosis was introduced, leveraging the capabilities of diverse algorithms. Specifically, six different algorithms known for their effectiveness in medical diagnosis were selected, including random forest, K-nearest Neighbors (KNN), Logistic Regression, Support Vector Machine (SVM), extreme gradient boosting (XGBoost), and decision tree. Extensive research has demonstrated the efficacy of these algorithms in various medical diagnosis tasks.

A novel ensemble consisting of three of these algorithms was developed to further enhance the robustness and accuracy of the model. By combining the predictions of the base models (SVM, XGBoost, random forest), the ensemble was formed using a voting technique. To achieve a more reliable and accurate outcome, the ensemble aggregates individual predictions and makes the final prediction based on a majority vote, thereby leveraging the collective knowledge of the models. The selection of the base models was carefully executed to create an ensemble that outperforms each individual model in isolation, achieving a superior performance.

### 2.5. Evaluation of Model Performance

To assess the performance of the developed machine learning algorithms, various statistical methods were employed. This included the calculation of performance metrics such as False Negatives (FN), True Positives (TP), True Negatives (TN), and False Positives (FP). These metrics were utilized to compute seven performance indicators: F1 score, precision, accuracy, recall, confusion matrix, AUC, and ROC. The confusion matrix provided an overview of the predicted outcomes. Furthermore, MAE was computed for the testing and training datasets.

## 3. Experimental Results

The dataset used in this study comprised a total of 2505 individuals, including 1310 females and 1195 males. It encompassed 23 features, thoroughly detailed in Table 1, along with additional analysis and information.

**Figure 1:**
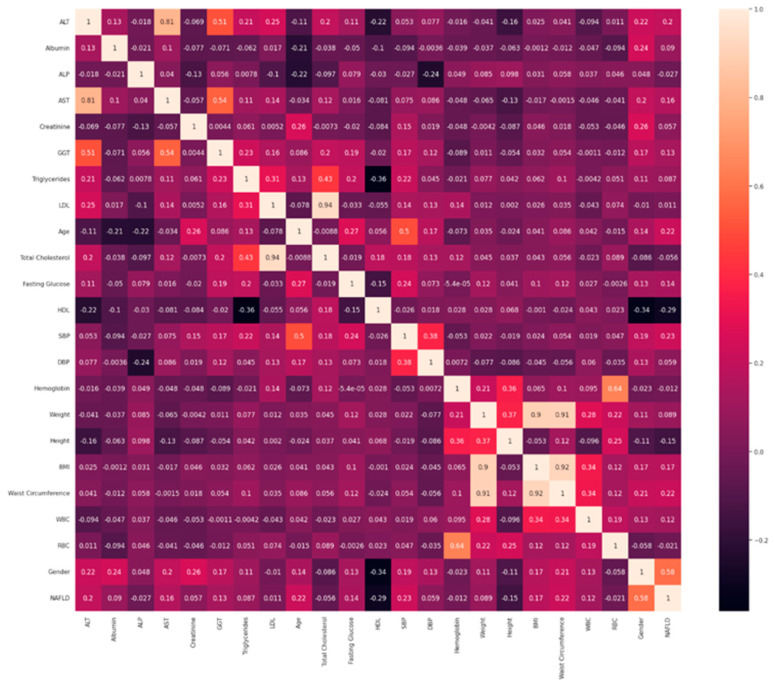
Heat map illustrating the interrelationships among various attributes.

After performing the necessary data preprocessing steps, such as elimination of duplicates, the treatment of missing values, and converting specific string attributes into numerical formats, the datasets underwent standardization. Following this, a comprehensive analysis of correlations was conducted, resulting in the production of heat maps that visually illustrated the relationships between the dataset’s features. This visualization is presented in Figure 1, providing valuable insights into the correlations between different variables.

Following a thorough analysis, each feature’s value was determined through embedded methods, enabling the ranking of features by their importance. The resulting feature importance rankings are presented in Table 2. Notably, the analysis revealed that gender holds the highest score among the features, indicating its significant impact.

The datasets utilized in this study were divided into a testing set, accounting for 20% of the data, and a training set, representing the remaining 80%. Table 3 displays the number of subjects in both the training and testing sets, along with the counts of individuals testing positive and negative for NAFLD.

The objective was to determine the most appropriate technique for predicting Non-Alcoholic Fatty Liver Disease (NAFLD), so six distinct machine learning techniques were developed and implemented. Additionally, an ensemble model was constructed to further enhance prediction accuracy. The performance of these models was then evaluated.

After calculating the HSI using a specific formula, the best cutoff (which is found to be 46.47) was determined to maximize AUC, accuracy. The primary goal of this evaluation was to assess the effectiveness of HSI in predicting NAFLD. Following this evaluation, the two sets of results were compared to determine their relative performance. The comparative results of these models are presented in Table 4.

To evaluate the performance of the implemented techniques, several evaluation metrics were considered. The evaluation metrics included accuracy, precision, recall, F1 score, and AUC. These metrics are detailed in Table 4.

**Figure 2:**
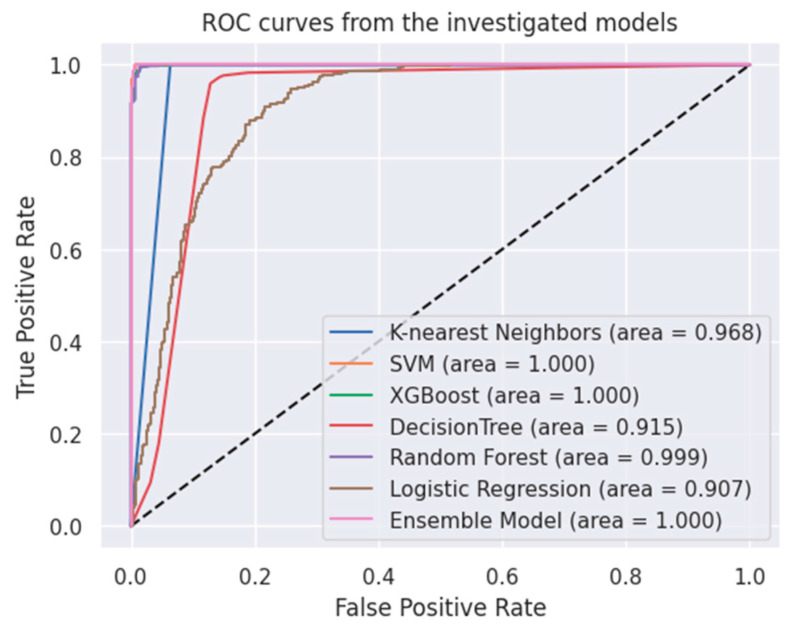
AUC and ROC curve obtained by Ensemble Model and Machine Learning Algorithms.

Figure 2 visually depicts AUC values and ROC curves acquired through employing the ensemble model and the machine learning algorithms. These metrics are crucial in assessing the performance and discrimination capabilities of the models. To compare the predicted values of the implemented techniques with the actual values, the confusion matrix was utilized. The confusion matrix, which outlines the true positives, true negatives, false positives, and false negatives, is detailed in Table 5. Additionally, MAE values for the testing and training datasets are provided in Table 6. These values are crucial indicators for assessing model performance and determining whether the model is overfitting to the training data. By comparing the MAE values, one can evaluate the generalization ability of the model.

**Figure 3:**
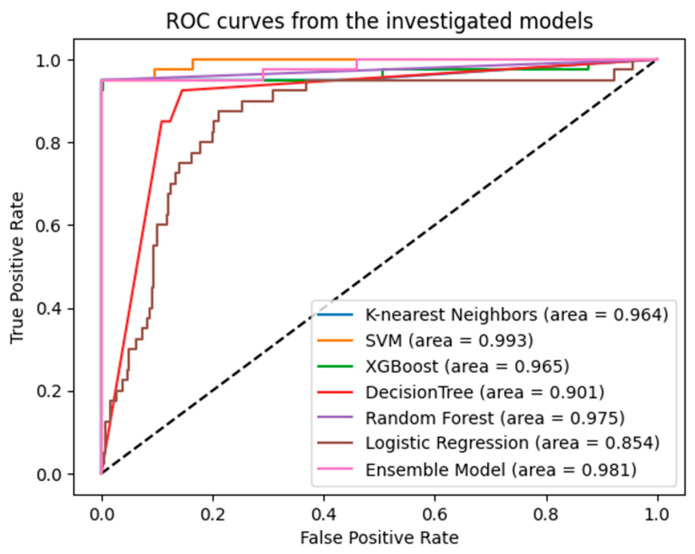
AUC and ROC curve obtained by Ensemble Model and Machine Learning Algorithms after applying SMOTEENN exclusively to the training set.

Table 7, Table 8, Table 9 and Table 10 and Figure 3 present the comparative results after exclusively applying SMOTEENN to the training set after dataset splitting.

## 4. Discussion

Based on the experimental results, the findings suggest that an ensemble model incorporating techniques random forest, XGBoost, and SVM, can serve as an effective tool for healthcare professionals and doctors in predicting NAFLD, utilizing affordable laboratory test data. This ensemble model demonstrates superior performance compared to this index, which has been utilized by doctors for evaluating and screening NAFLD.

Conventional statistical techniques have limitations in directly predicting NAFLD due to their reliance on selecting potential risk factors from data. To overcome these limitations, this study introduces machine learning techniques, which leverage statistical methods for data analysis and evaluation. Machine learning algorithms offer powerful tools for disease study and diagnosis, with the advantage of simultaneously considering multiple features without the need for variable selection. By applying machine learning techniques, this research aims to enhance NAFLD prediction by comprehensively analyzing diverse factors and their complex relationships, providing a data-driven and multivariate approach to improve diagnostic accuracy.

Furthermore, in our study, it was observed that HSI exhibited lower accuracy and AUC compared to the machine learning models utilized. This indicates that HSI was limited in its ability to accurately identify individuals with NAFLD, as it could only detect a small number of patients with the condition. Conversely, all of the machine learning models demonstrated higher F1 scores than HSI. This indicates that the machine learning models outperformed HSI in terms of precision and recall, achieving a better balance between correctly identifying true positives and minimizing false positives and false negatives. The higher F1 scores achieved by the machine learning models provide supporting evidence that they exhibited superior performance in predicting NAFLD compared to HSI.

SMOTEENN was also exclusively applied to the training set after the dataset was split. This adjustment was made to ensure the absence of potential data leakage and to ensure the robustness of our model evaluation. Our evaluation metrics, including accuracy, precision, recall, F1-score, and AUC, remained consistently high. Minor variations observed in certain metrics were within an acceptable range and did not compromise the overall performance of the model. Furthermore, the Mean Absolute Error (MAE) for both the training and testing datasets remained very low, indicating a strong fit of the model to the data. Confusion matrices were generated for both scenarios to provide a comprehensive assessment of our model’s performance. Given these results, confidence is asserted that data leakage did not occur. Additionally, it can be confirmed that no subject contributed data to both the training and test sets. In conclusion, applying SMOTEENN exclusively to the training set after splitting the dataset maintains the integrity of our findings while addressing potential concerns regarding data leakage. The consistency of our evaluation metrics reaffirms the robustness of our approach and the validity of our results.

Over the years, the utilization of machine learning techniques in disease prediction has been widely explored by numerous researchers. In a study conducted by Weidong Ji et al. [16], four machine learning algorithms were employed to predict Non-Alcoholic Fatty Liver Disease (NAFLD) using a dataset consisting of 304,145 adults. Among these four algorithms, XGBoost demonstrated the highest performance, with accuracy of 0.880 and AUC value of 0.951. Xu et al. [17] undertook a study to evaluate the optimal predictive clinical model for NAFLD using machine learning techniques. The study encompassed a dataset of 2,522 individuals who met the diagnostic criteria for NAFLD. Among the 11 different techniques employed, the best performance was observed, yielding an accuracy of 83%. Liu et al. [18] conducted a study aiming to explore the predictive capabilities of seven machine learning tools for NAFLD on 15,315 Chinese subjects. Among these models, the XGBoost model exhibited the highest accuracy, achieving a value of 0.795. This model demonstrated the best prediction ability among all the models constructed in the study, highlighting its superior performance in accurately diagnosing NAFLD.

Atsawarungruangkit et al. conducted a study aiming to create machine learning models for predicting Non-Alcoholic Fatty Liver Disease (NAFLD), utilizing data from the NHANES 1988–1994 dataset comprising 3235 participants, sourced from the National Center for Health Statistics (NCHS). Comparing the results, they found that the ensemble of random undersampling (RUS) boosted trees achieved the highest accuracy at 71.1%. Interestingly, a simpler model, referred to as “coarse trees,” outperformed this with an accuracy of 74.9% [25].

The findings of previous studies strongly support the effectiveness of machine learning tools in predicting NAFLD. These studies provide compelling evidence of the significant potential of machine learning algorithms in screening and identifying individuals at risk of NAFLD. The results affirm that machine learning techniques can be valuable tools in improving the accuracy and efficiency of NAFLD diagnosis. Consistent with the aforementioned studies, our own research corroborated the effectiveness of machine learning tools in NAFLD prediction. In our study, we developed an ensemble model using machine learning techniques, which achieved an impressive accuracy of 99% and an AUC of 100%. These results serve as further validation of the utility and efficacy of machine learning models in predicting NAFLD. Additionally, our findings highlight the potential of employing an ensemble approach to enhance the accuracy of NAFLD predictions. Together, the cumulative evidence from previous studies and our own research underscores the robustness and promising nature of machine learning tools in NAFLD prediction. These results emphasize the valuable contribution of machine learning algorithms in improving the screening and diagnosis of this prevalent liver disease. It is crucial to emphasize that while the ensemble model shows promising results in the accurate prediction of NAFLD, its effectiveness and integration into clinical practice require further research and validation studies. Nonetheless, the current findings indicate its potential as a valuable tool for healthcare professionals, utilizing affordable laboratory test data to aid in the diagnosis of NAFLD. To ensure its reliable implementation, additional research is warranted to validate its performance and establish its role in the healthcare setting.

Gender has emerged as a critically important factor in NAFLD, which is currently the most common liver disorder worldwide. Sexual dimorphism is evident in NAFLD, with notable disparities in both prevalence and severity based on gender. These differences are not solely influenced by sociocultural factors or lifestyle variations, but also attributed to biological disparities resulting from chromosomal makeup and sex hormone levels [26]. Numerous studies have demonstrated the impact of gender on NAFLD prevalence. For instance, a Japanese study conducted over 12 years found that the average prevalence of fatty liver in men was double that observed in women (26% vs. 13%). Interestingly, women exhibited a gradual increase in prevalence with age, whereas men demonstrated a relatively stable prevalence across all age groups. In the 70–79 age group, the prevalence of NAFLD was higher in females compared to males. Similarly, a study conducted in South China revealed a significantly higher prevalence of NAFLD in men compared to women below the age of 50 (22.4% vs. 7.1%). However, the prevalence reversed among individuals over the age of 50, with higher rates observed in women (27.6% vs. 20.6%). In humans, NAFLD predominantly affects men, while premenopausal women are equally protected from NAFLD and cardiovascular disease [27]. In our study, we conducted an analysis to determine the importance value of each feature and ranked them accordingly. Strikingly, the results indicated that gender exhibited the highest scores in terms of feature importance, reinforcing its significance in predicting NAFLD. These findings align with previous studies that have demonstrated the prominent role of gender in the prevalence and severity of NAFLD. Therefore, gender is a significant and influential feature in NAFLD. It plays a crucial role in the varying prevalence and severity of the disease. Understanding the mechanisms underlying gender disparities in NAFLD is crucial for developing targeted therapeutic interventions. Further research is needed to explore the complex interplay between gender, sex hormones, and NAFLD to optimize management strategies and improve patient outcomes.

This study has notable strengths that contribute to its robustness and generalizability. Firstly, it utilizes the NHANES dataset, which encompasses a diverse racial and ethnic background of individuals in the United States. The dataset is specifically designed to represent the non-institutionalized civilian population and includes oversampling of specific demographic groups, ensuring adequate representation. This approach enhances the study’s findings and minimizes potential bias. Additionally, this study leverages large-scale datasets for testing and training the algorithms, enabling a comprehensive evaluation across different models. Notably, an ensemble model is developed to enhance accuracy and optimize algorithm performance. These strengths collectively enhance the reliability and applicability of the study’s results.

Nevertheless, our study has certain limitations, notably the utilization of restricted datasets, the absence of clinical data, and the lack of a clinical trial. For future endeavors, our goal is to integrate further attributes pertaining to NAFLD, which will aid in the creation of more dependable and effective machine learning methods. Additionally, we strongly advocate for the implementation of a clinical trial to authenticate the efficacy of these techniques in real-world situations.

## 5. Conclusions

The results of our study highlight the effectiveness of the ensemble model, combining random forest, XGBoost, and SVM techniques, in accurately diagnosing NAFLD. The ensemble model achieved a remarkable performance, with an accuracy of 0.99 and an AUC of 1.00, indicating its high precision and reliability. Notably, our analysis identified gender as the most important feature in predicting NAFLD, further emphasizing its significance in this disease. In comparison to the other indices like HSI, our ensemble model demonstrated superior diagnostic capabilities and yielded substantially better results. These findings underscore the potential of the ensemble model for early detection and diagnosis of NAFLD, useful in screening.

## Figures and Tables

**Table 1:** Features Description.

Features	Range	Mean	Std	Range	Mean	Std
	NAFLD			Non-NAFLD		
Age (years)	13–80	51.94	19.93	12–80	45.38	20.60
BMI (kg/m^2^)	20.70–55.30	31.77	8.39	15.10–65.30	28.86	7.23
ALP (IU/L)	37–373	91.18	55.71	20–624	88.40	48.83
ALT (IU/L)	7–181	31.03	31.55	3–420	21.25	16.78
AST (IU/L)	12–77	25.94	13.07	6–198	21.44	11.91
DBP (mm Hg)	36–92	70.98	11.55	39–120	69.88	12.93
SBP (mm Hg)	98–176	130.16	17.11	78–216	122.47	19.02
GGT (IU/L)	9–137	38.42	32.20	4–414	28.23	33.05
Creatinine (mg/dL)	0.49–1.45	0.88	0.21	0.25–11.46	0.86	0.41
Triglycerides (mg/dL)	47–329	130.18	58.72	25–423	117.31	64.07
Fasting Glucose (mg/dL)	81–268	122.94	39.40	47–451	111.29	34.55
RBC (million cells/uL)	3.49–6.27	4.82	0.55	2.87–7.04	4.78	0.49
LDL (mg/dL)	46–178	109.88	34.08	18–357	107.59	35.62
HDL (mg/dL)	30–87	47.54	10.73	11–178	53.60	14.92
WBC (1000 cells/uL)	3.60–12.80	7.28	2.24	2.30–38.1	6.74	2.10
Total Cholesterol (mg/dL)	112–255	180.37	39.13	76–446	181.54	41.03
Weight (kg)	51.70–163	85.87	24.45	30.70–191.40	80.15	22.26
Height (cm)	144.80–187.20	164.24	9.32	134–195.30	166.32	10.07
Hemoglobin (g/dL)	8.90–17.20	14.07	1.67	6.40–18.70	14.05	1.54
Waist Circumference (cm)	76.40–151.90	106.14	18.48	59.20–169.50	97.74	17.75
Albumin (g/dL)	3.30–4.80	4.05	0.31	2.1–5.2	4.02	0.33

std: Standard deviation; GGT: *γ*-glutamyl transferase; AST: aspartate aminotransferase; ALT: alanine aminotransferase; ALP: Alkaline phosphatase.

**Table 2:** Rank of features importance.

Features	Rank
ALT	0.0302475
Albumin	0.03000044
ALP	0.03011238
AST	0.03231525
Creatinine	0.03287693
GGT	0.02722758
Triglycerides	0.04470876
LDL	0.03276697
Age	0.05981952
Total Cholesterol	0.03232777
Fasting Glucose	0.02749087
HDL	0.04328872
SBP	0.0530115
DBP	0.03660032
Hemoglobin	0.02463633
Weight	0.02756716
Height	0.03652343
BMI	0.03185026
Waist Circumference	0.04027026
WBC	0.02362244
RBC	0.02518571
Gender	0.27754989

**Table 3:** Number of subjects in the training and testing sets.

Dataset	NAFLD	Non-NAFLD
Training set	1907	1683
Testing set	467	444

**Table 4:** Comparison of Results: Ensemble Model and Machine Learning Algorithms and HIS index.

Machine Learning Techniques	Accuracy	Precision	Recall	F1-Score	Sensitivity	Specificity	Area under the Curve
Decision Tree	0.92	0.92	0.92	0.92	0.97	0.85	0.915
KNN	0.88	0.90	0. 87	0. 87	1	0.74	0.968
Random Forest	0.98	0.98	0.98	0.98	0.99	0.96	0.999
SVM	0.97	0.97	0.97	0.97	1	0.93	1.000
XGBoost	0.98	0.98	0.98	0.98	0.99	0.96	1.000
Logistic Regression	0.84	0.85	0.84	0.84	0.91	0.76	0.907
Ensemble Model	0.99	0.99	0.99	0.99	1	0.97	1.000
HSI index	0.74	0.77	0.62	0.68	0.62	0.72	0.73

**Table 5:** Numbers of TP, FN, TN, and FP cases of Ensemble Model and Machine Learning Algorithms.

Machine Learning Techniques	TP	TN	FN	FP
Decision Tree	454	381	13	63
Random Forest	466	430	1	14
K-nearest Neighbors classifier (KNN)	467	331	0	113
Support Vector Machine (SVM)	467	415	0	29
Logistic Regression	428	339	39	105
Extreme Gradient Boosting (XGBoost)	466	429	1	15
Ensemble Model	467	433	0	11

**Table 6:** MAE in testing and training phases of Ensemble Model and Machine Learning Algorithms.

Machine Learning Techniques	Testing	Training
Decision Tree	0.116	0.080
Random Forest	0.080	0.028
KNN	0.142	0.0
SVM	0.019	0.015
Logistic Regression	0.231	0.226
XGBoost	0.026	0.003
Ensemble Model	0.039	0.013

**Table 7:** Comparison of Results after applying SMOTEENN exclusively to the training set.

Machine Learning Techniques	Accuracy	Precision	Recall	F1-Score	Sensitivity	Specificity	Area under the Curve
Decision Tree	0.89	0.94	0.89	0.91	0.85	0.89	0.901
KNN	0.94	0.96	0.94	0.94	0.95	0.93	0.964
Random Forest	0.99	0.99	0.99	0.99	0.92	1	0.975
SVM	0.96	0.96	0.96	0.95	0.47	1	0.993
XGBoost	0.97	0.97	0.97	0.97	0.67	1	0.965
Logistic Regression	0.86	0.91	0.86	0.88	0.60	0.88	0.854
Ensemble Model	0.99	0.99	0.99	0.99	0.85	1	0.981
HSI index	0.74	0.77	0.62	0.68	0.62	0.72	0.73

**Table 8:** Numbers of TP, FN, TN, and FP cases after applying SMOTEENN exclusively to the training set.

Machine Learning Techniques	TP	TN	FN	FP
Decision Tree	34	411	6	50
Random Forest	37	461	3	0
K-nearest Neighbors classifier (KNN)	38	431	2	30
Support Vector Machine (SVM)	19	461	21	0
Logistic Regression	24	407	16	54
Extreme Gradient Boosting (XGBoost)	27	461	13	0
Ensemble Model	34	461	6	0

**Table 9:** MAE in testing and training phases after applying SMOTEENN exclusively to the training set.

Machine Learning Techniques	Testing	Training
Decision Tree	0.11	0.11
Random Forest	0.00	0.00
KNN	0.08	0.00
SVM	0.04	0.00
Logistic Regression	0.15	0.14
XGBoost	0.02	0.00
Ensemble Model	0.02	0.00

**Table 10:** Number of subjects in the training and testing sets after applying SMOTEENN exclusively to the training set.

Dataset	NAFLD	Non-NAFLD
Training set	1881	1588
Testing set	40	461

## Data Availability

The NHANES dataset, the Centers for Disease Control (CDC) of the United States as a part of the National Health and Nutrition Examination Survey (NHANES), available online at https://wwwn.cdc.gov/nchs/nhanes/continuousnhanes/default.aspx?BeginYear=2017 (accessed on 15 February 2020).
